# Mechanical and Tribological Characterization of a Dental Ceromer

**DOI:** 10.3390/jfb11010011

**Published:** 2020-02-21

**Authors:** Mariana Santos, Ana Sofia Coelho, Anabela Baptista Paula, Carlos Miguel Marto, Inês Amaro, José Saraiva, Manuel Marques Ferreira, Pedro Antunes, Eunice Carrilho

**Affiliations:** 1 Institute of Integrated Clinical Practice, Faculty of Medicine, University of Coimbra, 3000-075 Coimbra, Portugal; anasofiacoelho@gmail.com (A.S.C.); anabelabppaula@sapo.pt (A.B.P.); mig-marto@hotmail.com (C.M.M.); ines.amaros@hotmail.com (I.A.); ze-93@hotmail.com (J.S.); eunicecarrilho@gmail.com (E.C.); 2 Institute for Clinical and Biomedical Research (iCBR), Area of Environment, Genetics and Oncobiology (CIMAGO), Faculty of Medicine, University of Coimbra, 3000-548 Coimbra, Portugal; m.mferreira@netcabo.pt; 3 CNC.IBILI Consortium, Faculty of Medicine, University of Coimbra, 3000-548 Coimbra, Portugal; 4 Centre for Innovative Biomedicine and Biotechnology (CIBB), University of Coimbra, 3000-548 Coimbra, Portugal; 5 Institute of Experimental Pathology, Faculty of Medicine, University of Coimbra, 3000-548 Coimbra, Portugal; 6 Institute of Endodontics, Faculty of Medicine, University of Coimbra, 3000-075 Coimbra, Portugal; 7 Department of Mechanical Engineering, Centre for Mechanical Engineering, Materials and Processes (CEMMPRE), University of Coimbra, 3030-788 Coimbra, Portugal; pedro.antunes@dem.uc.pt; 8 Itecons—Institute of Research and Technological Development in Construction, Energy, Environment and Sustainability, Rua Pedro Hispano, 3030-289 Coimbra, Portugal

**Keywords:** ceromer, ceramics, composite resin, mechanical properties, thermocycling

## Abstract

Background: Indirect restorations using composites with ceramic fillings can be an alternative to ceramic veneering and direct composite restorations for the treatment of posterior teeth. The aim of this study was the evaluation of the mechanical and tribological properties of a ceromer. Materials and Methods: Sixty specimens were produced and divided into two groups: one control group not submitted to thermocycling (n = 20) and one test group submitted to 5000 cycles of thermocycling (n = 40). The studied parameters were microhardness, surface roughness and the coefficient of friction (scratch test). Results: The ceromer exhibits a reduction of polymerization shrinkage, higher wear, and fracture resistance than the composite resins. The studied ceromer presented good mechanical properties, even after being submitted to thermocycling. Roughness was the property most affected, increasing 25.8%, microhardness decreased by 10.5% and the coefficient of friction increased by 4.2%. Conclusions: In certain situations, ceromers can be an alternative to composite resins and ceramics, providing an aesthetic, conservative and longevity option.

## 1. Introduction

The continuous evolution of materials and techniques used in dentistry, especially adhesive systems, and restorative materials, aims to provide materials with excellent aesthetic and mechanical properties, capable of restoring function, shape, contour, and color, as well as restoring tooth resistance [1,2]. The application of nanotechnology in the field of biomaterials has driven the development of dental materials, namely composite resins, improving their mechanical and biological properties [3,4].

Both teeth and restorative materials are subject to oral cavity conditions such as humidity, load cycles, temperature variation, bacteria, external agents and contact geometry [5], which makes it challenging to find a material that can withstand the mechanical, thermal and chemical stresses [6] without damaging the structures of the oral cavity while maintaining high longevity. The degradation and erosion of resin composites occur more easily when incomplete polymerizated and/or when the teeth isolation during treatments is not achieved, due to degradation processes caused by oxidation [7]. Material loss due to wear may be the result of physiological factors, such as friction during chewing, pathological factors such as bruxism, and prophylactic factors, such as abrasion which occurs during brushing [8]. Restoration wear is one of the main causes of failure of composite resins in posterior teeth [5].

However, the main disadvantage of direct composite resin rehabilitation is the high polymerization contraction of these materials [9,10], which may be from 2% to up to 5% [2]. Marginal infiltration is the most common clinical consequence in situations where there is decompensation of forces at the tooth-restoration interface, exceeding the adhesion force. When forces related to contraction stress are inferior to the adhesive force, cusp flexion and postoperative sensitivity may occur [2,10,11]. Since polymerization shrinkage remains the main problem of composite resins, the use of these materials should be limited to small cavities to increase their clinical success [10,12].

Ceramic rehabilitation is a treatment that is often offered to patients. This treatment has many advantages, such as the aesthetic factor (enamel-like translucency), the possibility of characterizing both the internal and external surfaces, and the high clinical success rate [11]. However, these materials have a very high modulus of elasticity, which can lead to fractures [13], due to the difficulty in absorbing occlusal forces. This can result in the transfer of stress to the interface of the adhesion material [14]. Furthermore, their high wear resistance can lead to antagonist tooth deterioration [15].

To overcome all the disadvantages of direct composite resins and ceramics, resinous materials with ceramic charge, known as ceromers, appeared about 15 years ago [11]. Ceromers contain inorganic micro-hybrid particles, surrounded by a photopolymerizable organic matrix [16,17]. These materials have a monomer conversion rate in the order of 98% [11]. This factor improves the mechanical properties, particularly the marginal integrity and the color of the restoration-tooth interface [10]. Additionally, its lower modulus of elasticity, more similar to the modulus of elasticity of dentin (when compared to ceramic), allows a better distribution of forces when in function, providing a deformation very similar to that of the natural tooth when subjected to intra-oral stress [17]. Further advantages are now described for the use of ceramic-reinforced resin materials such as high wear and abrasion resistance, good aesthetics, low fracture potential, high flexural strength, reduced antagonist tooth wear, maintenance of marginal integrity and easy intra-oral repair [2,15,16,18,19,20]. Ku et al. [21] reported that the main advantage of using this material is the possibility of fabrication of crowns with enough mechanical strength to resist occlusal forces, maintaining aesthetics and marginal integrity [21].

However, some disadvantages are associated with these materials, such as having a lower hardness than enamel and long-term persistent roughness, which may result in restoration staining, marginal staining, bacterial plaque retention and aesthetic alteration [2,15,16,18,19,20].

This study aimed to evaluate the mechanical and tribological properties of a ceromer, before and after thermocycling. According to the manufacturer, this material contains approximately 80% (by weight) nanoceramic particles bound to the resin matrix. Ceramic particles are composed of three different types of ceramic fillers that reinforce the highly cross-linked polymer matrix. The charges are composed of non-aggregated particles of silica (20 nanometres) and zirconia (4 to 11 nanometres) and aggregate particles of zirconia (4 to 11 nanometres) and silica (20 nanometres).

## 2. Materials and Methods

Sixty specimens of resinous restorative material with ceramic charge, 3M ESPE Lava Ultimate CAD/CAM Restorative^®^ (3M ESPE, Neuss, Germany), were molded in the following dimensions in millimeters: 4 × 6 × 12 (thickness × width × length). The superficies of the specimens were treated with silicon carbide (SiC) sandpaper with increasing particle size (P1000, P2000), with continuous water flow, for 20 s, using the LaboPol-5 (Struers A/S, Ballerup, Denmark) machine to reduce roughness. Subsequently, the specimens were subjected to an ultrasound bath for 10 min to remove any impurity that might have arisen during polishing and then were numbered and measured with a Mitutoyo Co. digital calliper (Kawasaki, Japan). Each specimen was further weighed on a precision A&D Semi-Micro Analytical Balance GH 202 scale (Tokyo 170-0013, Tokyo, Japan).

The specimens were divided into two groups: one control group (n = 20), not subjected to thermocycling, and one test group (n = 40) submitted to 5000 cycles of thermocycling for 60 s with a temperature variation of 5 to 55 °C. The control group was only used for the scratch test.

The mechanical properties of the material were studied before and after the thermocycling cycles. The Vickers microhardness test was carried out and surface roughness was also evaluated. The tribological sliding indentation test was chosen. The scratch specimens produced by the tribological test were observed in a scanning electronic microscope (SEM).

### 2.1. Surface Microhardness Analysis

Surface microhardness was determined by the Vickers test method; a Struers Duramin durometer (Struers, Balleruo, Denmark) was used according to standard ASTM E384-10 [22]. The Vickers microhardness value, HV, was determined, after measuring the diagonals left by the indenter in the samples. Due to the low loads of the Vickers hardness measurements, several tests were done with different indentation loads in order to ensure that the indentation appearance wasn’t changed, nor an indentation size effect appeared. Thus, for the selected load the microhardness values were independent of indentation size (Kick’s law [23]).

A 200 g load (1.962 N) was applied due to the viscoelastic nature of the material for 40 s and ten indentations were made on the surface of each block. Measurements of each indentation were made according to the standard test method for micro-indentation material hardness.

### 2.2. Surface Roughness

Surface roughness was determined using a surface roughness evaluation machine, Mitutoyo Surftest 402 Surface Tester (Mitutoyo Co., Kawasaki, Japan), according to ISO 4288 [24] standard pattern. The analyzed parameters are presented in Table 1.

The arithmetic mean of the evaluation profile (R_a_) corresponds to the arithmetic average of the absolute values of the profile heights over the evaluation length, where *c* is the base length. The maximum height of the profile (R_z_) corresponds to the average of the maximum height of the profile peaks, Z_p_, with the maximum depth of the profile values, Z_v_, included in the sample length, lr. The R_max_ corresponds to the highest-profile roughness value during the measurement path, which is the highest value of R_z_. The mean square deviation of the evaluated profile (R_q_) corresponds to the root mean square average of the profile heights over the evaluation length, where c is the base length.

The maximum height of the roughness profile (R_t_) is defined as the sum of the highest of the peak height of the profile, Z_p_, with the highest of the valley depths of the profile, Z_v_, within the evaluation length, ln. The maximum height of the profile peaks (R_p_) corresponds to the distance from the maximum peak to the midline.

Five measurements were performed on each specimen, uniformly and at a constant speed, along the surface. The specimens were placed on a metal support, with the same orientation of their length. The extent of surface measurement was from 0.8 to 4 mm, as indicated in Table 2, for a R_a_ between 0.1 and 2 μm.

### 2.3. Tribological Characterization

The study of the material wear resistance was performed through the sliding indentation test, which allows the applied load to be varied and, consequently, the frictional force. The characteristics of this test allow unidirectional contact, and the possibility of measuring, with precision, the normal and tangential loads along the test; ability to observe the scars generated during the test allowing to associate to each point the contacting loads, in both normal and tangential directions.

To determine the coefficient of friction, a scratch test was performed using a computed numerically controlled machine (CNC) in order to allow a precise displacement and load control, (Mikron, Agno, Switzerland), according to ASTM G 171-13 norm [25]. This equipment allows precise control of the force applied and the deformation of the specimen. A rounded, 50 μm, conical (60°) tungsten carbide tip, horizontally scribed the blocks at a constant velocity of 0.5 mm/s, with a linearly increasing charge overtime of 0 to 50 N, producing scratches of 5 mm in length. When advancing through the specimen, the indenter creates a compression zone in the material and a traction zone as it travels the predetermined length. The roughness will be the first to come into contact with the indenter, increasing the friction and, consequently, separation of particles from the matrix may occur.

**Figure 1:**
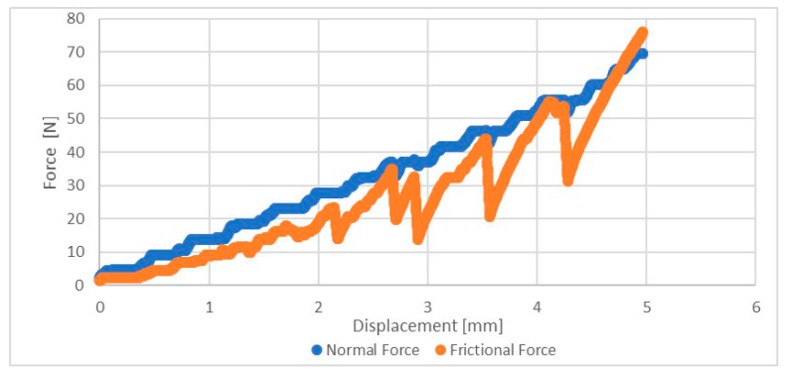
Friction and normal forces due to the displacement.

The friction force increases linearly with the normal force applied by the indenter, as exemplified in Figure 1.

The slope of the line represents the coefficient of friction, μ, which is obtained by representing the frictional force as a function of the normal force. The surface observations of the sample were performed with a Philips XL 30 (Eindhoven, The Netherlands). Despite the calculation of the coefficient of friction, the wear surface analysis was only qualitative.

### 2.4. Statistical Analysis

The results were initially evaluated in a descriptive way, for which adequate statistics were calculated. For the description of the quantitative variables, the mean and the standard deviation were chosen. In addition to the descriptive analysis, we used parametric and non-parametric statistical tests, according to the verification of the assumptions. For the quantitative variables, the normality assumption was verified through the Shapiro–Wilk test. The Wilcoxon–Mann–Whitney test was used to evaluate differences between the means of two groups dependent on quantitative variables. The level of significance adopted throughout the statistical analysis was 5%. The software used in data processing was IBM^®^SPSS^®^ v.24.0 (IBM Corporation, Armonk, NY, USA). The whole process of statistical analysis was blind.

## 3. Results

### 3.1. Weight

No differences were identified between the weight of the specimens evaluated before (0.4467 ± 0.0385 g) and after (0.4480 ± 0.0385 g) 5000 cycles of thermocycling (Z = −1.923; *p* = 0.054).

### 3.2. Microhardness

**Figure 2:**
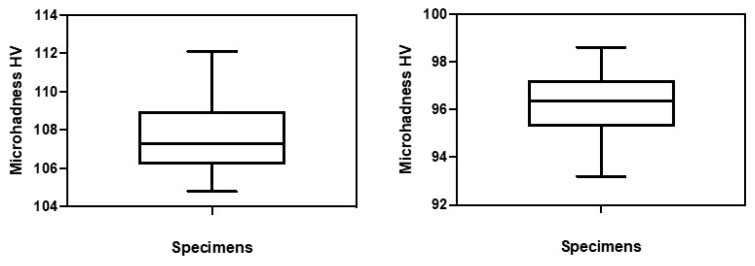
Average initial and final microhardness values and respective standard deviations of the specimens.

The mean initial and final microhardness values and respective standard deviation for each specimen are shown in Figure 2.

**Figure 3:**
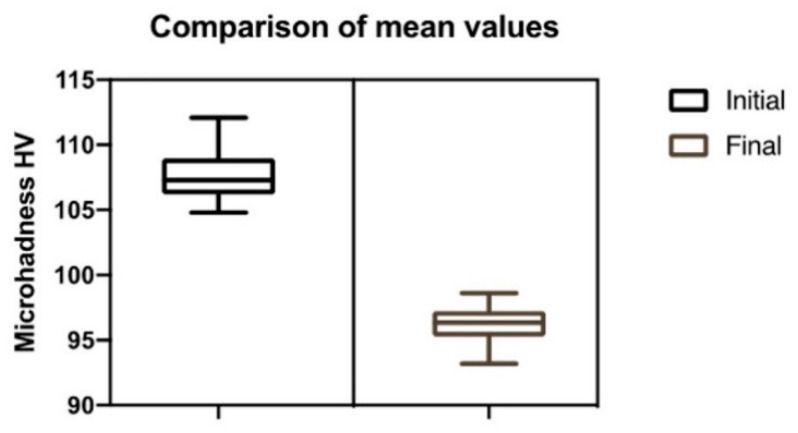
Comparison of the arithmetic means of the group before and after thermocycling.

In Figure 3 the initial and final microhardness values (after 5000 cycles of thermocycling) of the evaluated ceromer are shown. There was a 10.5% decrease in the microhardness of the material after 5000 cycles of thermocycling. The average initial microhardness was 107.66 ± 4.84 HV and the mean final microhardness value was 96.34 ± 3.22 (Z = −5.511, *p* < 0.001).

### 3.3. Roughness

The roughness results were represented by the different variables considered in the study: R_a_, R_z_, R_max_, R_q_, R_t_, and R_p_.

Statistically significant differences were found between the initial and final evaluations for the R_z_ (Z = −2.742; *p* = 0.005), R_max_ (Z = −3.549; *p* < 0.001), R_t_ (Z = −3.381; *p* = 0.001) and R_p_ (Z = −2.267; *p* = 0.025). No significant differences were found for the values of R_a_ (Z = −1.540; *p* = 0.125) and R_q_ (Z = −1.806; *p* = 0.071).

**Figure 4:**
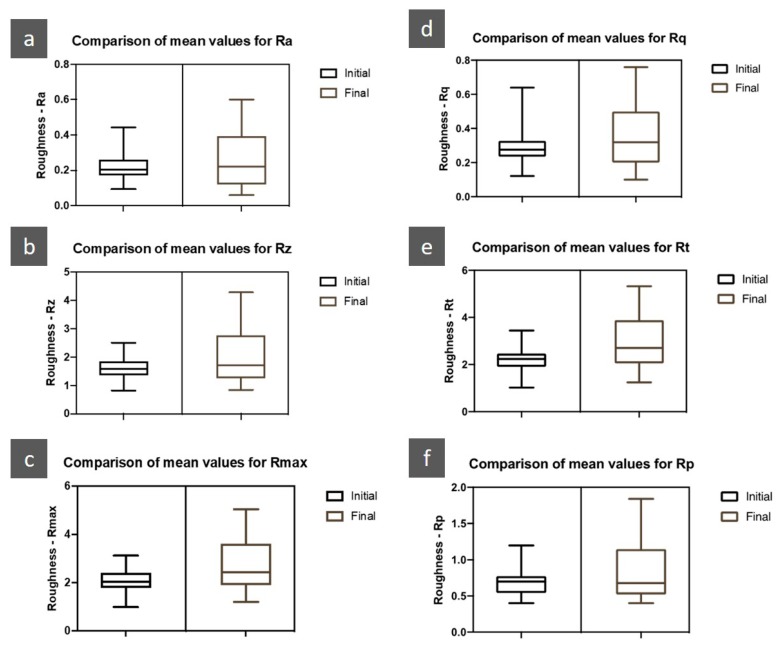
Comparison of the arithmetic means of the roughness (initial and final) for R_a_ (**a**), R_z_ (**b**), R_max_ (**c**), R_q_ (**d**), R_t_ (**e**), R_p_ (**f**).

Figure 4 shows the initial and final R_a_, R_z_, R_max_, Rq, R_t_ and R_p_ values (after 5000 thermocycling cycles).

There was a 19.3% increase in the value of R_a_. The mean value of the initial R_a_ was 0.22 ± 0.07 μm and the mean value of the final Ra was 0.26 ± 0.15 μm. There was a 27.7% increase in the value of R_z_. The mean value of the initial R_z_ was 1.61 ± 0.39 μm and the mean value of the final R_z_ was 2.05 ± 0.61 μm. There was a 32.4% increase in the R_max_ value. The mean value of initial the R_max_ was 2.08 ± 0.49 μm and the mean value of final the R_max_ was 2.75 ± 1.08 μm. There was a 20.3% increase in the value of R_q_. The mean value of the initial R_q_ was 0.29 ± 0.10 μm and the mean value of the final R_q_ was 0.35 ± 0.18 μm. There was a 33.7% increase in the value of R_t_. The average value of the initial R_t_ was 2.21 ± 0.54 μm and the mean value of the final R_t_ was 2.95 ± 1.16 μm. There was a 21.4% increase in the value of R_p_. The mean value of the initial R_p_ was 0.69 ± 0.17 μm and the mean value of the final R_p_ was 0.84 ± 0.39 μm.

**Figure 5:**
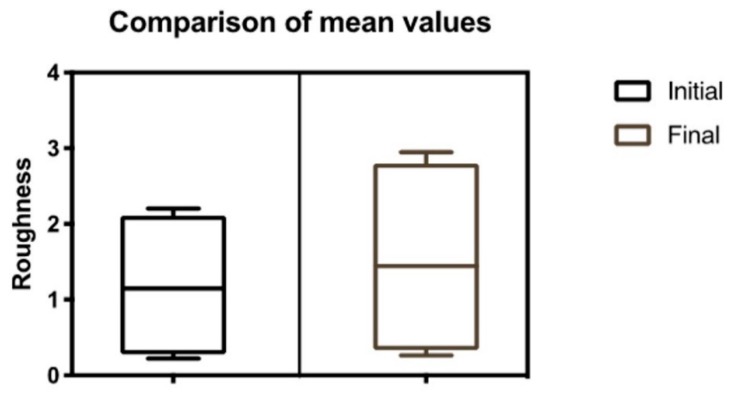
Comparison of arithmetic means for all roughness parameters.

Figure 5 shows a comparison of the arithmetic averages of roughness parameters analyzed. Overall, there was an increase in roughness of 25.8%.

### 3.4. Sliding Indentation Test

The results related to the friction coefficient of the studied material, determined by the Scratch Test, are shown in Table 3.

**Figure 6:**
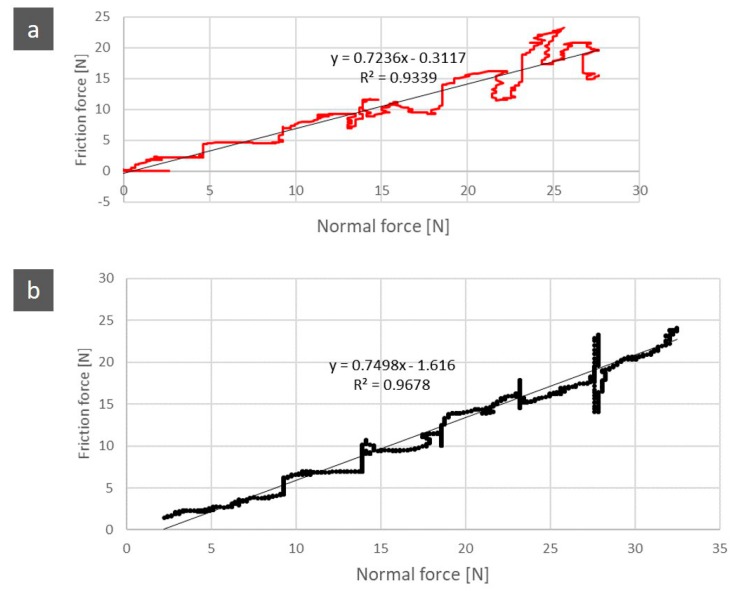
Relation between the normal force and the friction force of the control group (**a**) and test group (**b**), with the coefficient of friction of 0.72 and 0.75 respectively.

Figure 6 shows the relationship between the normal force and the frictional force, with its coefficient of friction. Between the control group and the test group, there was an increase of 4.2%.

**Figure 7:**
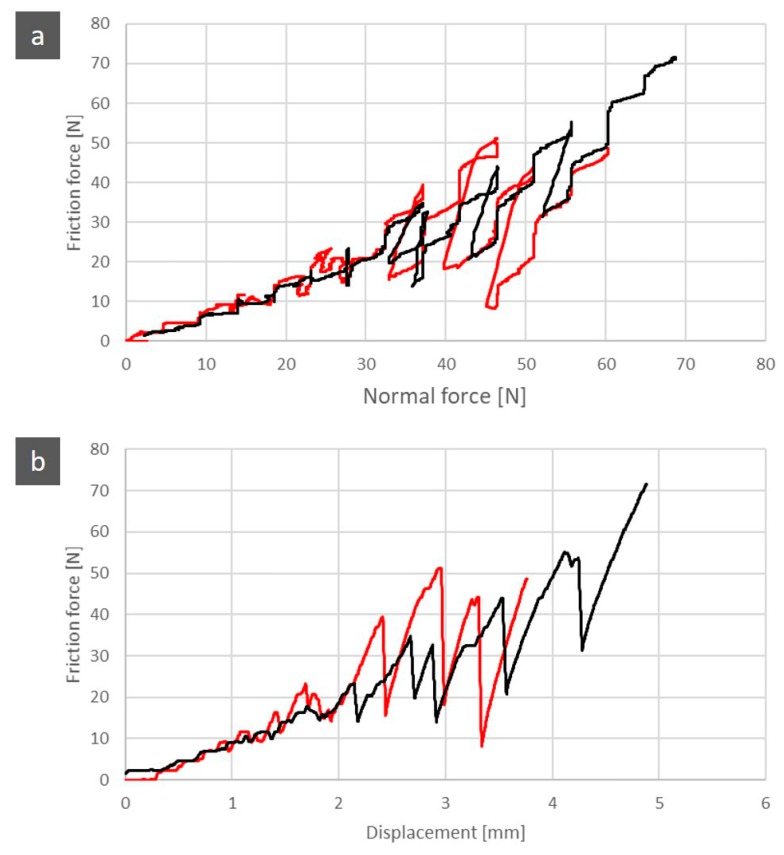
Comparison between the normal force and the friction force of control and test groups (**a**). Comparison of the friction force between the control and test groups (**b**).

Figure 7 is the graphical representation of the coefficient of friction comparison and the frictional force between the control group and the test group. There was a greater oscillation in the friction force of the control group than in the test group.

**Figure 8:**
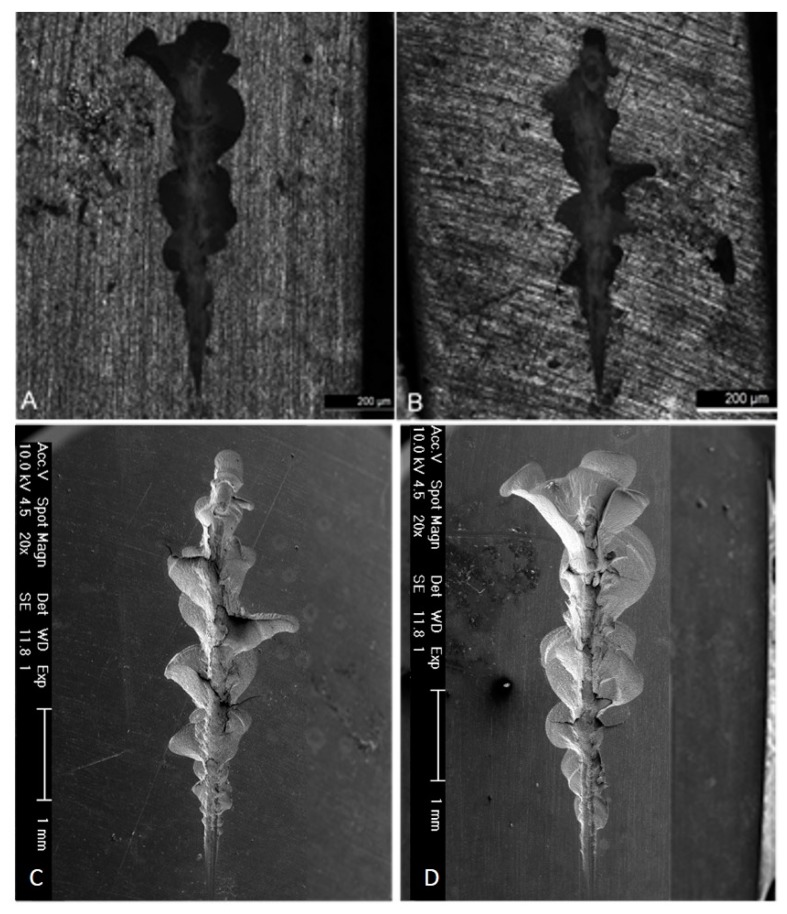
Wear observation on non-polarized lens. (**A**) Control group; (**B**) Test group, and SEM imagens for, (**C**) Control group; (**D**) Test group.

The presented analysis concerning the evolution of friction force and the comparison of the coefficient of friction is corroborated by the images obtained from both optical and electronic microscopes. In Figure 8, wear marks are shown on a 200 μm scale.

**Figure 9:**
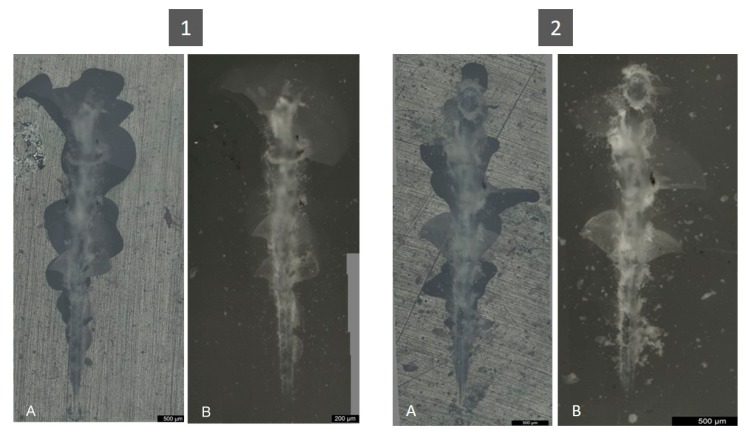
Observation of wear suffered by a specimen of the control group (1) and test group (2): (**A**) Non-polarized lens; (**B**) Polarized lens.

**Figure 10:**
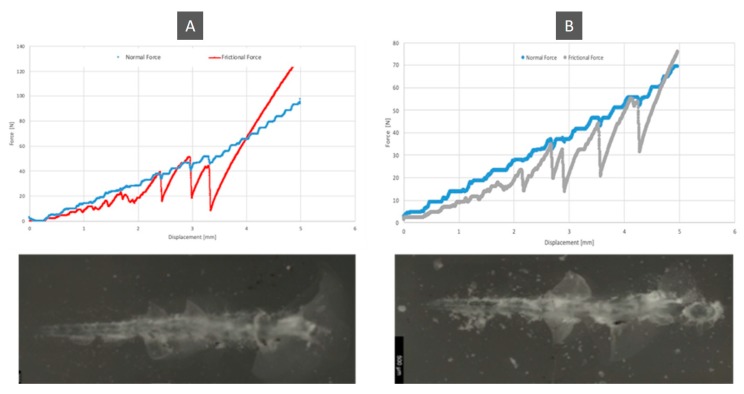
Wear observation obtained for control and test groups compared to normal force and frictional force. (**A**) Control group; **(B)** Test group.

Figure 9 shows the wear scars that the indenter left on specimens from both groups. The wear marks represented correlate with the graphs of the friction force (Figure 10). The control group had larger and more irregular fractures. For the same initial indenter displacement, the surface of the specimen showed greater damage in the control group than in the test group. The control group had larger and more irregular fractures.

**Figure 11:**
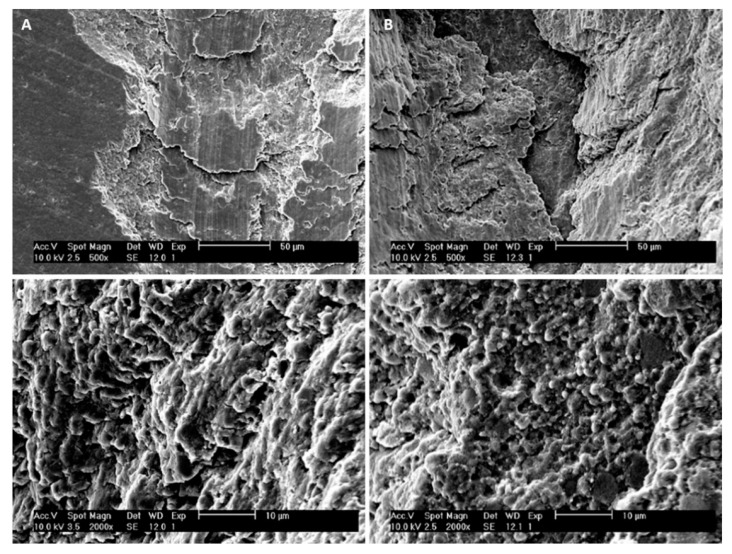
Detailed wear images observation obtained for: (**A**) Control group (500× top image and 2000× bottom image); (**B**) Test group (500× top image and 2000× bottom image).

Figure 10 shows the wear scars along with the friction force and normal force plot that the indenter left on both specimens. Relating these graphics to the wear scar produced by each indenter on the antagonist allows us to establish a relationship between the observed morphology features and the values of the loads that occurred in those specific places. From the analysis of the previous graphs assembled along with the SEM pictures scar surface of the ceromer, it was possible to find the exact location of the fractures produced by the increasing loading of the indenter. This gives the perception of the severity of the damage. Figure 11 shows the various details of the scratch test for control and test groups.

## 4. Discussion

In this experimental study, several mechanical parameters of the 3M ESPE Lava Ultimate CAD/CAM Restorative^®^ ceramics (3M ESPE, Neuss, Germany) were tested. The initial microhardness of the material was 107 Vickers (1049 MPa), which after 5000 cycles of thermocycling reduced for 96 Vickers (941.5 MPa). These values are closer to the values of microhardness of dentin, which is around 1000 MPa, than the microhardness of enamel, which is around 4000 MPa [26]. The microhardness values of the non-reinforced composite resins are around 90 Vickers (883 MPa) [27]. Reinforcement with ceramic particles positively influences the mechanical properties of the composite resins and, even after aging, these properties are better than the properties of the non-reinforced composite resins.

The degradation that takes place in the restorations, whether these are composite resins or composite resins reinforced with ceramic particles, is because, when in contact with water (and, in the case of the oral cavity, with saliva), the diffusion of water molecules occurs in the polymer matrix, which degrades the siloxane bonds, compromising the physical and mechanical properties of the material. This produces a decrease in the hardness and modulus of elasticity of the restorations over time [6,28].

Roughness has been indicated as the main disadvantage resulting from the use of ceromers, as this increases over time (13,18). In the present study, the roughness parameters that varied significantly after 5000 thermocycling cycles were R_z_, R_max_, R_t_, and R_p_. The maximum roughness parameter (R_max_) provides data on the deterioration of the vertical surface of the specimens. Thus, its increase is associated with an increase in peaks and roughness on the surface of the restoring material [6]. The total roughness depth parameter (R_t_) measures the distance between the highest peak and the deepest valley, within the total evaluation length, which also supports a significant increase in R_max_. The mean square root of the height of profiles (R_q_) and the arithmetic mean of the absolute values of the profile heights (R_a_) do not define the shape of the irregularities [29], which may explain the fact that there were no statistically significant differences in these parameters. In general, in the present study, the roughness of the specimens increased by 25.8% after the aging of the specimens; this corroborates the results of previous studies, which refer to the significant increase in the surface roughness of the ceromers [10,11,13,18]. However, the present study would benefit from an increase in the number of specimens, since the standard deviation of the initial measurements intersected the arithmetic means of the final measurements.

The coefficient of friction increased after thermocycling, and a variation of the frictional force between the test group and the control group was identified. This has been observed in materials that have hard reinforcing particles [6] and therefore resist the advancement of the indenter, causing a decrease in their roughness. The larger the size of the reinforcing particles, the higher the coefficient of friction and consequently the higher the contact forces, which can increase the volume of material removed by wear [6].

The damages that were presented in the direction transversal to the displacement occurred via subsurface fractures, due to the increase of the coefficient of friction (or the friction force), when reaching high local values (state of higher tension), taking into account that the voltage distribution obeys the Hertz theory [30] and that the tangential component of the applied load increases the local voltage in the subsurface. Thus, with the increase of the frictional force and consequently of the stress in the subsurface of the material, this voltage distribution induced surface faults below the contact area through a fatigue process and, consequently, the propagation of this failure to the surface occurred. This explains the larger fractures in the test group compared to the control group. However, the matrix of this material is resinous and, in addition to the behavior of the reinforcing material, it is also important to consider the possible wear that the resin matrix may suffer, compromising its behavior.

Increased roughness appears to indicate that thermocycling affects the matrix, not the particles. Surface roughness influence on the wear behavior can be explained by the materials strength which is determined by its surface roughness and the inner structure of the material. An increase in the surface roughness may cause a larger stress concentration than the one produced by the surface roughness in combination with the surface flaws [31]. If a material has low roughness, when in contact with other materials is less prone for cracks to appear. This is possible to observe (Figure 9), that the initial scar path on the thermocycled specimens, the ones with higher values of roughness parameters, present a larger wear scar then the control material group, for the same test conditions. The smother the material surface more difficult is to the cracks to appear, and therefore if the material remains smoother the restoration will be long lasting. The matrix can both retract with respect to the particles and become softer [5], which justifies the greater resistance of the test group to scratches, with more subsurface fractures, which were expected to occur in the zones of higher wear, and oscillations of the force of friction. The more premature appearance of subsurface fractures and their greater extend indicates that the material in the control group is more fragile than the test group, which was also confirmed by microhardness analysis. For the control group, which had a higher reinforcement load and lower toughness, the indenter encountered higher resistance, changing the friction force in relation to the normal force. In the test group, as toughness increased, the slopes of the friction force and normal force were similar.

## 5. Conclusions

Ceromers can be an aesthetic alternative to restorations of teeth with great destruction, showing clinical longevity, with a low fracture rate and low incidence of caries recurrence. The fracture limit of ceromers is superior to the occlusal forces exerted, which, together with t its elasticity modulus similar to dentin. These results support the possibility that these materials can be used in patients with parafunctional habits, however, future clinical studies must be performed to determine the clinical performance of these materials. In areas where aesthetics is fundamental (anterior sector), ceramics may be a more viable option.

The studied ceromer, Lava Ultimate CAD/CAM Restorative^®^ ceramics, proved to be a good material for complex restorations since it maintains excellent mechanical properties even after aging.

## Figures and Tables

**Table 1:** Roughness parameters analyzed.

Roughness Parameters	
R_a_	Roughness Average
R_z_	Average Maximum Height of the Profile
R_max_	Maximum Roughness Depth
R_q_	RMS Roughness
R_t_	Maximum Height of the Profile
R_p_	Maximum Profile Peak Height

**Table 2:** Tabulated values according to standard pattern EM ISO 4288 for sampling length selection.

Periodic Profiles	Non-Periodic Profiles		Cut-off	Length of Sample Evaluation
R_sm_ (mm)	R_z_ (µm)	R_a_ (µm)	*λ*_c_ (mm)	lr/ln (mm)
>0.013 and <0.04	<0.1	<0.02	0.08	0.08/0.4
>0.04 and <0.13	>0.1 and <0.5	>0.02 and <0.1	0.25	0.25/1.25
>0.13 and <0.4	>0.5 and <10	>0.1 and <2	0.8	0.8/4
>0.4 and <1.3	>10 and <50	>2 and <10	2.5	2.5/12.5
>1.3 and <4	>50 and <200	>10 and <80	8	8/40

**Table 3:** Average value of the coefficient of friction.

	Control Group	Test Group
µ	0.72	0.75
